# Dynamics of Dispersive Measurements of Flux-Qubit States: Energy-Level Splitting Connected to Quantum Wave Mechanics

**DOI:** 10.3390/nano13172395

**Published:** 2023-08-23

**Authors:** Jeong Ryeol Choi

**Affiliations:** School of Electronic Engineering, Kyonggi University, Yeongtong-gu, Suwon 16227, Gyeonggi-do, Republic of Korea; choiardor@hanmail.net

**Keywords:** flux qubit, superconducting quantum interference device, energy level, Hamiltonian, unitary transformation

## Abstract

Superconducting flux qubits have many advantages as a storage of quantum information, such as broad range tunability of frequency, small-size fabricability, and high controllability. In the flux qubit–oscillator, qubits are connected to SQUID resonators for the purpose of performing dispersive non-destructive readouts of qubit signals with high fidelity. In this work, we propose a theoretical model for analyzing quantum characteristics of a flux qubit–oscillator on the basis of quantum solutions obtained using a unitary transformation approach. The energy levels of the combined system (qubit + resonator) are analyzed in detail. Equally spaced each energy level of the resonator splits into two parts depending on qubit states. Besides, coupling of the qubit to the resonator brings about an additional modification in the split energy levels. So long as the coupling strength and the tunnel splitting are not zero but finite values, the energy-level splitting of the resonator does not disappear. We conclude that quantum nondemolition dispersive measurements of the qubit states are possible by inducing bifurcation of the resonator states through the coupling.

## 1. Introduction

Nanomaterial-based quantum information devices may play a crucial role in next-generation quantum science and engineering. Among such devices, superconducting nanocircuits [1,2,3,4,5,6] are simple quantum systems that can potentially be used for controlling universal quantum gates in quantum computers. Typically, these circuits are composed of superconducting qubits framed with Josephson junctions that allow large-scale integrability. Owing to recent advances in quantum nanotechnology, these have become one of the main research subjects in quantum information science such as quantum computing and quantum simulation. Through the developments in fabricating quantum gates, not only the controllability of single-qubits but also the capability of coupling between them in a way that the circuit allows decoherence-free information processes [7,8,9] is highly desired.

As is well known, a qubit is a two-level system that serves as a basic unit for storing quantum information in quantum computers. A peculiar property of qubits, which is clearly distinguished from that of classical bits, is that they can be a superposition of the two states as well as one of both states according to the principle of quantum mechanics. Several kinds of qubits materialized on the basis of superconducting circuits are flux qubits, charge qubits, and phase qubits [4,5]. In this work, we focus on flux qubits because they can be competitively applied as quantum information storages with the availability of real-time readout of their states [10,11,12]. While flux qubits exhibit usual quantum properties such as discrete energy levels, entanglement, quantum interference, and superposed quantum states, it is possible to control them to preserve their high coherence.

A flux qubit coupled to an oscillator (resonator) can be a potential resource for encoding and processing quantum information in quantum computation [8]. For this reason, the interaction of a two-level system with an oscillator has become a highly emerging research topic over the years. The operation of a qubit is actually the same as a single atom that has a large electric dipole moment coupled to the cavity mode of the microwave photons. Quantum interference that takes place in superconducting devices is in fact a coherent superposition of probability fields. The feasibility of many experimental operations with flux qubit–oscillators upon theoretical backgrounds has been demonstrated thus far. For instance, recording Berry phases for geometric operations [13,14], preparation of squeezed states [15,16], non-demolition readout of qubit information [17,18], and adaptation of multi-qubit algorithms [19,20,21] are possible.

Quantum dynamics of superconducting flux qubit–oscillators is investigated in this work, based on a theoretical model for analyzing them. In particular, the energy splitting in the resonator, which appears due to coupling of a qubit on it, is examined in detail. The harmonic oscillator connected to a flux qubit plays the role of measuring the qubit states, whereas it is usually composed of a superconducting quantum interference device (SQUID). Because the formula of the Hamiltonian for a qubit–oscillator is complicated in most cases, direct evaluation of quantum solutions is very difficult. For this reason, we adopt a unitary transformation approach which is a powerful auxiliary method for managing a system described by a complicated Hamiltonian matrix. Unitary transformation with the matrix can be performed without loss of the Hermitian nature of the matrix. The additive and multiplicative relationships between the operators are kept through such transformations. The Hamiltonian can be simplified via the transformation, facilitating the investigation of various quantum properties of the qubit–oscillator system on the basis of complete quantum solutions.

## 2. Description of the Hamiltonian

Flux qubits can be applied in a scalable manner to quantum information science owing to the fact that not only can their frequency be broadly tuned, but qubits also exhibit high relaxation time and strong anharmonicity. Thanks to the anharmonicity in a multi-qubit system, we can rapidly control pulses without significant frequency crowding [22]. For the detailed design and fabrication of flux qubits, refer to Refs. [10,20,22,23].

Let us consider a flux qubit coupled to a SQUID oscillator [8]. This coupling exhibits many interesting effects concerning the experiments of cavity quantum electrodynamics and ion/atom traps, which facilitate the generation and probe of nonclassical states important in quantum devices. Further, it is possible to produce entangled states between qubits from such coupling, which are crucial in quantum-state engineering for quantum information processing [24,25]. This entanglement is necessary in quantum computing and can be enhanced by the increase in relaxation time of the oscillator and/or the coupling strength [8].

By coupling qubits to the SQUID oscillator via current distribution in the shared regions, an additional term which indicates an interaction appears in the Hamiltonian. Considering this, the overall Hamiltonian of this system is given by

(1) $$\hat{H}={\hat{H}}_{q}+{\hat{H}}_{\mathrm{sq}}+{\hat{H}}_{q-\mathrm{sq}},$$

where ${\hat{H}}_{q}$ is the qubit Hamiltonian, ${\hat{H}}_{\mathrm{sq}}$ is the SQUID circuit Hamiltonian, and ${\hat{H}}_{q-\mathrm{sq}}$ is the Hamiltonian that represents the interaction between them. Let us see the details of each term in Equation (1). The qubit–oscillator system can be explained via a spin-boson model that adopts an effective spectral density [26,27]. Two levels in the flux qubit are distinguished by the clockwise/anticlockwise directions of the controllable persistent currents [28] corresponding to the spin-up/spin-down states respectively. These two states are coupled by tunneling with the tunnel splitting Δ that has the dimension of frequency. According to this, the qubit Hamiltonian for the system is given by

(2) $${\hat{H}}_{q}=-\hbar \left(\varepsilon {\hat{\sigma }}_{z}+\delta {\hat{\sigma }}_{x}\right)/2,$$

where ${\hat{\sigma }}_{x}$ and ${\hat{\sigma }}_{z}$ are Pauli matrixes with the basis of spin-up/spin-down, ℏε is the qubit maximum persistent current, and δ=2πΔ. The approximate value of ε is given by $\varepsilon ≃I_{p}\Phi_{0}\left(\gamma_{q}-\pi \right)/\left(\hbar \pi \right)$, where $\Phi_{0}=\pi \hbar /e$ is the flux quantum, $\gamma_{q}$ is the superconductor phase across overall junctions and $I_{p}$ is the qubit maximum persistent current. In many cases of flux qubits, the junctions are three-fold [8]. The energy splitting between the ground and the excited states in the qubit is given by $E_{d}^{q}=h\Delta_{L}$ where $\Delta_{L}$ is the Larmor frequency of the qubit. The Larmor frequency is determined by the two coefficients of Equation (2), i.e., $\Delta_{L}=\sqrt{\Delta^{2}+\epsilon^{2}}$ where ϵ=ε/(2π). The minimum energy splitting is hΔ that is given in the case ε≃0 [29].

The SQUID acts as a harmonic oscillator of an LC circuit coupled, in general, to an Ohmic bath. The overall inductance of the SQUID circuit is given by $L_{t}=L_{j}+L_{\mathrm{se}}$ where $L_{j}$ is the Josephson inductance of the junctions and $L_{\mathrm{se}}$ is self-inductance of the SQUID and shunt lines. Then, the angular frequency of the SQUID circuit can be represented as

(3) $$\omega_{p}={\left[L_{t}C_{\mathrm{sh}}\right]}^{-1/2},$$

where $C_{\mathrm{sh}}$ is the shunt capacitance. The Hamiltonian for an ideal SQUID circuit is given by

(4) $${\hat{H}}_{\mathrm{sq}}=\hbar \omega_{p}\left({\hat{a}}^{†}\hat{a}+1/2\right),$$

where we have neglected the interaction of the circuit with Ohmic environment [30].

Finally, the interaction Hamiltonian is of the form

(5) $${\hat{H}}_{q-\mathrm{sq}}=\lambda {\hat{\sigma }}_{z}\left(\hat{a}+{\hat{a}}^{†}\right),$$

where λ is the coupling strength. Lots of interesting effects associated with qubit–oscillator systems described by Equation (1) can also be applied to a wide range of physics besides quantum computation. For instance, their applicable fields include quantum dots in photonic crystals [31] and single dipole atoms coupled to cavity microwave photons [32]. The qubit signals in the SQUID environmental noise suffer decoherence and dissipation depending largely on the coupling strength between the qubit and the SQUID [30].

The detection characteristics, including noise effects, of a dc-SQUID inductively coupled on the qubit mainly depend on λ. Hence, we need to modulate λ in an effective way depending on the aim of the research. If we want a good readout resolution of qubit states, a strong coupling is necessary. On the other hand, in the case where we need to reduce the negative effects of the environment, we must keep the coupling strength small [30]. In this work, we choose the latter case because it is favorable for quantum nondemolition measurements of qubit states.

Regarding the general quantum mechanics, let us express the annihilation operator in the form $\hat{a}=\sqrt{\Omega /\left(2\hbar \right)}\hat{q}+i\hat{p}/\sqrt{2\hbar \Omega },$ where $\hat{p}=-i\hbar \partial /\partial q$ and $\Omega =L_{t}\omega_{p}$. Then, as we know, its hermitian adjoint ${\hat{a}}^{†}$ is the creation operator. Now, Equation (1) can be written in a matrix form as

(6) $$\hat{H}=\left(\begin{array}{cc} \hat{X}+\hat{Y} & D\\ D & \hat{X}-\hat{Y} \end{array}\right),$$

where D=−ℏδ/2 and the operators are $\hat{X}={\hat{p}}^{2}/\left(2L_{t}\right)+L_{t}\omega_{p}^{2}{\hat{q}}^{2}/2$ and $\hat{Y}=\sqrt{2\Omega /\hbar }\lambda \hat{q}-\hbar \varepsilon /2.$ By solving the Schrödinger wave equation with this Hamiltonian in the subsequent section, we derive energy eigenvalues and the corresponding wave functions, which are fundamental in the research of quantum properties of the system.

## 3. Results and Discussion

### 3.1. Unitary Transformation Approach

To investigate quantum dynamics of the system, it is necessary to see the eigenvalue problem of the Hamiltonian given in Equation (6). We put the Schrödinger solutions (wave functions) of the system as

(7) $$\psi_{n}\left(q,t\right)=\left(\begin{array}{c} \psi_{n,+}\left(q,t\right)\\ \psi_{n,-}\left(q,t\right) \end{array}\right).$$

Here, $\psi_{n,+}\left(q,t\right)$ corresponds to the wave of spin-down whereas $\psi_{n,-}\left(q,t\right)$ corresponds to spin-up. Since Equation (6) is a complicated matrix form, the direct evaluation of these wave functions may be very difficult. Special mathematical techniques are necessary in order to find their analytical forms. To overcome this difficulty, we simplify the Hamiltonian using a two-step unitary transformation. Our first step of transformation is focused on diagonalizing the Hamiltonian, and the second step is on transforming the Hamiltonian to a most simplified one. For these purposes, we introduce suitable unitary operators $\hat{U}$ and ${\hat{U}}_{\pm }$ as

(8) $$\hat{U}=\frac{1}{\sqrt{N}}\left(\begin{array}{cc} \hat{Y}+\sqrt{{\hat{Y}}^{2}+D^{2}} & D\\ D & -\left(\hat{Y}+\sqrt{{\hat{Y}}^{2}+D^{2}}\right) \end{array}\right),$$

(9) $${\hat{U}}_{\pm }=\exp\left(\frac{i}{\hbar }d_{\pm }\hat{p}\right),$$

where

(10) $$N=2\left[{\hat{Y}}^{2}+D^{2}+\hat{Y}\sqrt{{\hat{Y}}^{2}+D^{2}}\right],$$

(11) $$d_{\pm }=\pm \sqrt{2\omega_{p}/\left(L_{t}\hbar \right)}\lambda \varepsilon /\left(\omega_{L}\omega_{p,\pm }^{2}\right),$$

while $\omega_{L}=\sqrt{\varepsilon^{2}+\delta^{2}}$ and

(12) $$\omega_{p,\pm }^{2}=\omega_{p}^{2}\pm \frac{4\omega_{p}\lambda^{2}}{\hbar^{2}}\left(\frac{1}{\omega_{L}}-\frac{\varepsilon^{2}}{\omega_{L}^{3}}\right).$$

At this stage, we consider the case of a weak coupling strength:

(13) λ/ℏ≪ε,δ.

In fact, this is a crucial requirement in order to preserve quantum coherence in quantum computation devices [33]. A quantum nondemolition measurement of the qubit states can be realized by using the oscillator-type resonator under the limit given in Equation (13) [9,34]. The production of Landau–Zener interference [35] and the securing of amplitude spectroscopy [36] are also possible under this condition. In addition, weak coupling allows to generate Fock, dressed, and Floquet states [33,37], which are hard to realize by other means [37].

For an arbitrary matrix $\hat{M}$, the unitary transformation rule with a unitary operator $\hat{U}$ is given by ${\hat{M}}^{\prime }=\hat{U}\hat{M}{\hat{U}}^{†}$. Now, we carry out the two-step unitary transformation using Equation (8) in the first and Equation (9) in the second step: we use $\hat{U}$ in the first transformation and then ${\hat{U}}_{\pm }$ are used in the second transformation. From this, we have the final transformed Hamiltonian as (for detailed methods of transformation, see Appendix A)

(14) $${\hat{H}}_{\pm }^{\prime \prime }=\frac{{\hat{p}}^{2}}{2L_{t}}+\frac{1}{2}L_{t}\omega_{p,\pm }^{2}{\hat{q}}^{2}+H_{\pm },$$

where $H_{\pm }=E_{\pm }^{q}+E_{\pm }^{\mathrm{add}}$ with $E_{\pm }^{q}=\pm \hbar \omega_{L}/2,$ and

(15) $$E_{\pm }^{\mathrm{add}}=-L_{t}\omega_{p,\pm }^{2}d_{\pm }^{2}/2.$$

**Figure 1:**
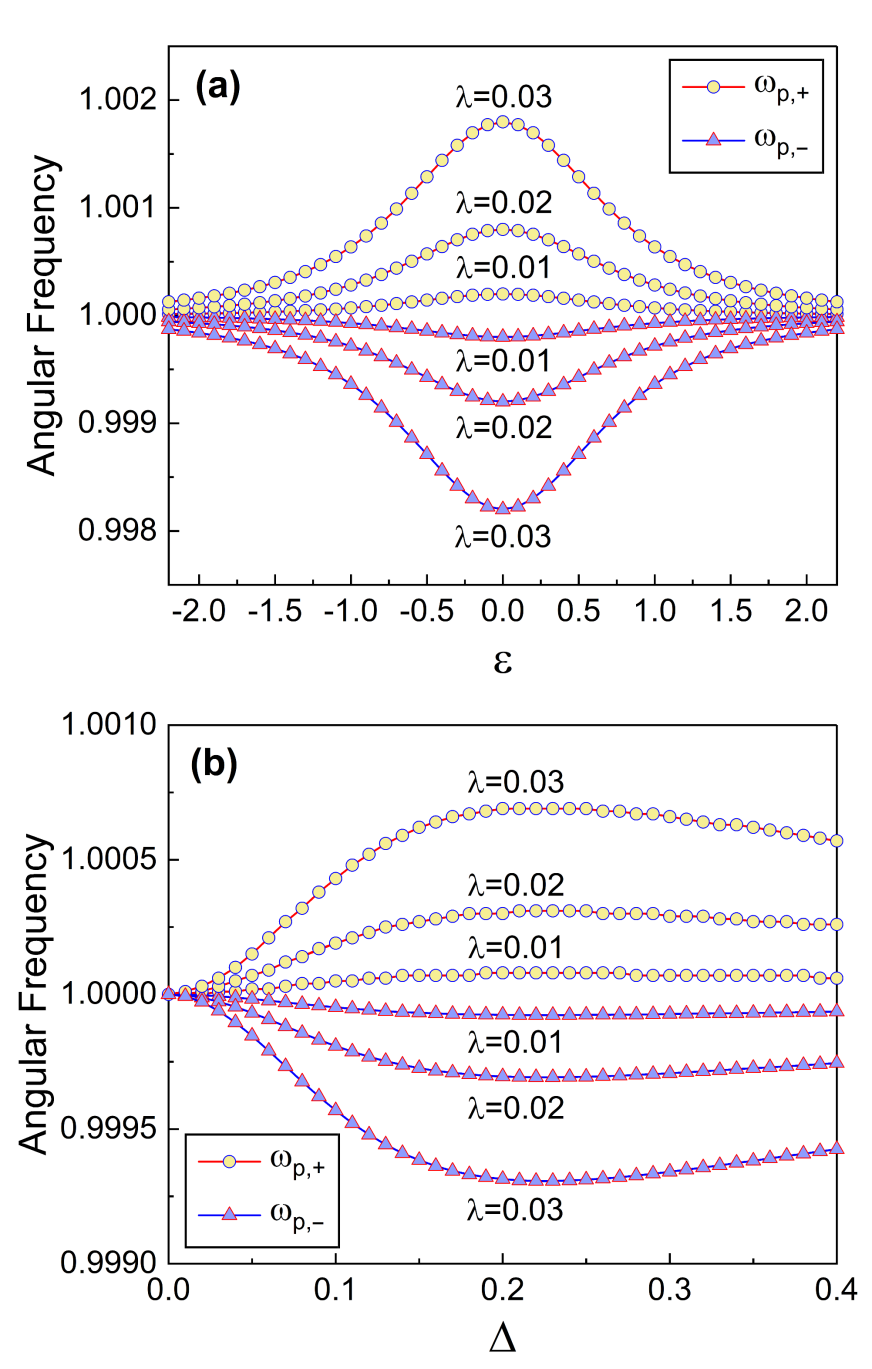
Modified angular frequency $\omega_{p,\pm }$ as a function of ε (**a**) and Δ (**b**) for several values of λ. δ=1 for (**a**) and ε=1 for (**b**). We have used $L_{t}=1$, $C_{\mathrm{sh}}=1$, and ℏ=1.

The transformed Hamiltonian is thus described by the modified angular frequencies $\omega_{p,\pm }$ of which modifications are deeply related to the connection of the oscillator to the qubit. That is, such modifications are determined by the coupling constant λ (see Equation (12)). Frequency modifications imply that the frequency splits into two parts, whereas the dependencies of that splitting on ε and Δ are shown in Figure 1. We can confirm from Figure 1a that the difference between the split frequencies, $\omega_{d}=\omega_{p,+}-\omega_{p,-}$, is largest when ε is zero. A detailed evaluation using Equation (12) at ε=0 gives $\omega_{d}=\omega_{p}\left(A_{+}-A_{-}\right)$ where $A_{\pm }={\left[1\pm 4\lambda^{2}/\left(\hbar^{2}\delta \omega_{p}\right)\right]}^{1/2}$. Whereas $\omega_{d}$ increases as λ grows, $\omega_{d}$ becomes zero in the limit λ→0 as expected. Figure 1b exhibits that $\omega_{d}$ is zero when Δ=0 and it increases as Δ augments and reaches maximum at a specific value of Δ: that value (Δ) is about 0.23 in the case of the graphs in Figure 1b.

The merit of the unitary transformation methods is their potentialities regarding mathematical simplifications of the problem as shown up to now. The versatile properties of unitary operators can also be used for other purposes in this field. For instance, one can carry out quantum computing through properly prepared quantum states by implementing sequences of unitary gates. Moreover, the time evolution of a closed system can also be described via a unitary transformation.

### 3.2. Analysis of Energy-Level Splitting

We can write the eigenvalue equations for the transformed Hamiltonian in the form ${\hat{H}}_{\pm }^{\prime \prime }|\psi_{n,\pm }^{\prime \prime }\rangle =E_{n,\pm }|\psi_{n,\pm }^{\prime \prime }\rangle$. Then, based on the evaluations in Section 3.1, the energy eigenvalues are given by

(16) $$E_{n,\pm }=E_{n,\pm }^{\mathrm{SHO}}+E_{\pm }^{q}+E_{\pm }^{\mathrm{add}},$$

where $E_{n,\pm }^{\mathrm{SHO}}$ are the eigenvalues of the Hamiltonian of the simple harmonic oscillators (SHOs) of which angular frequencies are $\omega_{p,\pm }$: $E_{n,\pm }^{\mathrm{SHO}}=\left(n+1/2\right)\hbar \omega_{p,\pm }$. $E_{n,\pm }^{\mathrm{SHO}}$ and $E_{\pm }^{q}$ are associated with the SQUID energies and the qubit energy levels respectively, while $E_{\pm }^{\mathrm{add}}$ are additional energies that appear due to the coupling of the qubit to the SQUID.

**Figure 2:**
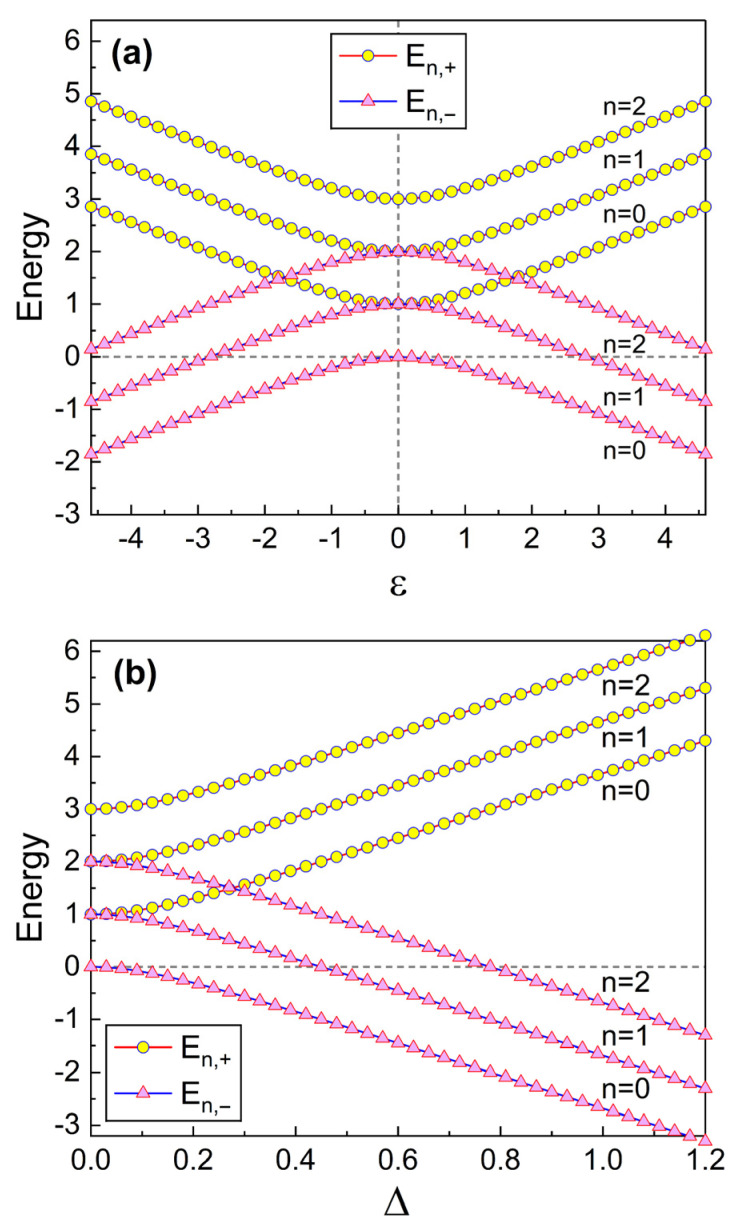
Energy eigenvalues $E_{n,\pm }$ of the combined system for the first three quantum numbers *n* plotted as a function of ε (**a**) and Δ (**b**). The value of δ is 1 for (**a**) and the value of ε is 1 for (**b**). We have used $L_{t}=1$, $C_{\mathrm{sh}}=1$, λ=0.01, and ℏ=1.

It is apparent by inspecting Equation (16) that $\omega_{p,\pm }$ play major roles in determining $E_{n,\pm }$, where $\omega_{p,\pm }$ are represented in terms of ε and Δ as can be confirmed from Equation (12). We have plotted $E_{n,\pm }$ in Figure 2 for a more detailed analysis regarding this. The dependence of $E_{n,\pm }$ on ε can be seen from Figure 2a and on Δ from Figure 2b for the first three lowest quantum numbers *n*. We can examine the overall energy differences $E_{n,d}=E_{n,+}-E_{n,-}$ of the combined system from these graphics. Figure 2a reveals that these differences are smallest when ε=0 and increase as |ε| becomes larger.

The detailed evaluation of $E_{n,d}$ using Equation (16) in the limit ε=0 gives $E_{n,d}=\hbar \left[\delta +\omega_{d}\left(n+1/2\right)\right]$. The first term ℏδ is the usual energy gap in the qubit. On the other hand, the second term, $\hbar \omega_{d}\left(n+1/2\right)\left[\equiv \Delta E_{n}\right]$, is an additional energy difference that originated from the splitting of the modified angular frequency, $\omega_{p,\pm }$, in the SQUID resonator, which was previously analyzed from Figure 1. Notice that $E_{\pm }^{\mathrm{add}}$ do not contribute to $E_{n,d}$ in this case since $E_{\pm }^{\mathrm{add}}=0$ when ε=0. Because $\omega_{d}$ increases as λ grows, $\Delta E_{n}$ are large for a large λ. When ε=0, the additional energy difference $\Delta E_{n}$ with a specific quantum number *n* is directly proportional to *n* and becomes small as the actual qubit energy gap, hΔ, increases. However, $\Delta E_{n}$ vanish in the case λ→0 because $\omega_{d}=0$ in that limit. Hence, we see that, if the coupling has been removed (λ=0), the energy differences are reduced to $E_{n,d}=\hbar \delta$ which are the same value as the pure energy gap of the qubit. By the way, for both cases ε≠0 and ε=0, the energy-level splitting of the resonator does not disappear provided that the coupling strength and the tunnel splitting are not zero but finite values. From Figure 2b, we can confirm that, as Δ grows from zero, $E_{n,d}$ increase nearly monotonically from the smallest value.

**Figure 3:**
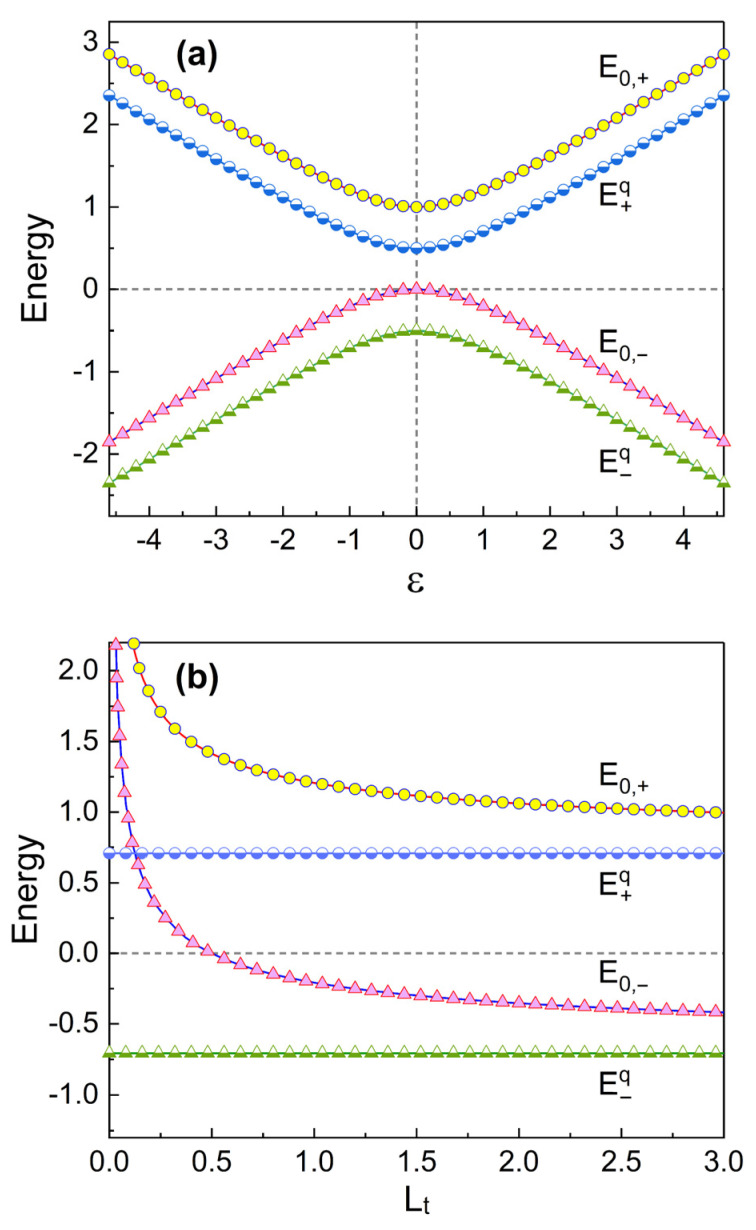
Comparison of energy eigenvalues $E_{0,\pm }$ with the qubit energy $E_{\pm }^{q}$ plotted as a function of ε (**a**) and $L_{t}$ (**b**). The value of $L_{t}$ is 1 for (**a**) and the value of ε is 1 for (**b**). We have used $C_{\mathrm{sh}}=1$, λ=0.01, δ=1, and ℏ=1.

We have compared $E_{0,\pm }$ with the qubit energy levels $E_{\pm }^{q}$ in Figure 3. Figure 3a exhibits that $E_{0,+}$ ($E_{0,-}$) is larger than $E_{+}^{q}$ ($E_{-}^{q}$) by a constant energy which is given almost regardless of ε. Roughly speaking, $E_{0,\pm }$ are larger than $E_{\pm }^{q}$ by an amount of the zero point energy of the SQUID resonator. Figure 3b shows that $E_{0,\pm }$ (and, consequently, $E_{n,\pm }$ with an arbitrary number *n*) decrease as $L_{t}$ increases. $E_{0,\pm }$ also decrease as $C_{\mathrm{sh}}$ becomes large (not shown here). $E_{n,\pm }$ are determined by $\omega_{p,\pm }$, which decrease as $L_{t}$ and/or $C_{\mathrm{sh}}$ increase (see Equation (16) with Equations (15), (12) and (3)). However, the qubit energy levels are irrelevant to $L_{t}$ and $C_{\mathrm{sh}}$.

**Figure 4:**
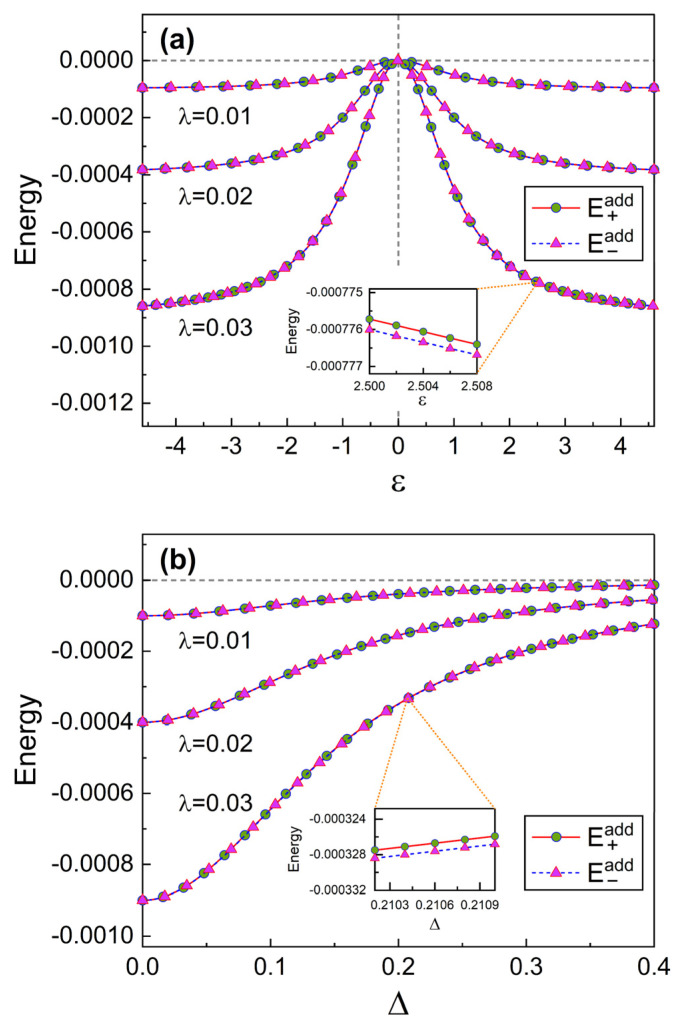
Additional energies $E_{\pm }^{\mathrm{add}}$ as a function of ε (**a**) and Δ (**b**) for several different values of λ. The value of δ is 1 for (**a**) and the value of ε is 1 for (**b**). We have used $L_{t}=1$, $C_{\mathrm{sh}}=1$, and ℏ=1. The insets are enlargement of the indicated part in the graphics.

In fact, the additional energies, $E_{\pm }^{\mathrm{add}}$, in Equation (16) are very small in the case of weak coupling. The analysis of their detailed characteristics may be interesting though. We see from Figure 4 that the absolute values of $E_{\pm }^{\mathrm{add}}$ increase as the coupling strength λ becomes large. Because the energies $E_{\pm }^{\mathrm{add}}$ take place due to the coupling of the qubit on the SQUID resonator, their dependence on the coupling strength λ is very sensitive. Consequently, if we disconnect the qubit from the resonator by setting λ=0, $E_{\pm }^{\mathrm{add}}$ vanish. In Figure 4, the behaviors of $E_{+}^{\mathrm{add}}$ and $E_{-}^{\mathrm{add}}$ look quite the same as each other. Strictly speaking however, they are slightly different and this can be confirmed from the insets in Figure 4 drawn based on Equation (15).

In the presence of the microwave drive while measuring the qubit, the SQUID-based resonator would exhibit nonlinearity. This nonlinearity may allow highly sensitive bifurcation readout of qubit states, leading possibly to achieving a direct nondemolition readout joined with the fast and efficient qubit measurements [17].

### 3.3. Quantum Wave Mechanics

If we do not regard the last term in Equation (14), the individual Hamiltonians ${\hat{H}}_{\pm }^{\prime \prime }$ just correspond to those of SHOs with the frequencies $\omega_{p,\pm }$, whose whole quantum solutions are well known. The wave functions in the original system can be obtained from the inverse transformation of the wave functions associated to the final transformed Hamiltonian which is Equation (14). That transformation leads to (see Appendix B)

(17) $$\psi_{n}\left(q,t\right)={\hat{U}}^{†}\left(\begin{array}{c} \phi_{n,+}^{\prime }\left(q\right)\exp\left[i\theta_{n,+}\left(t\right)\right]\\ \phi_{n,-}^{\prime }\left(q\right)\exp\left[i\theta_{n,-}\left(t\right)\right] \end{array}\right),$$

where $\phi_{n,\pm }^{\prime }\left(q\right)$ are functions of *q* and $\theta_{n,\pm }\left(t\right)$ are phases, which are of the forms

(18) $$\phi_{n,\pm }^{\prime }\left(q\right)={\left(\frac{\Omega_{\pm }}{\hbar \pi }\right)}^{1/4}\frac{1}{\sqrt{2^{n}n!}}H_{n}\left[{\left(\frac{\Omega_{\pm }}{\hbar }\right)}^{1/2}\left(q-d_{\pm }\right)\right]\exp\left(-\frac{\Omega_{\pm }}{2\hbar }{\left(q-d_{\pm }\right)}^{2}\right),$$

(19) $$\theta_{n,\pm }\left(t\right)=-\omega_{p,\pm }t\left(n+1/2\right)-\hbar^{-1}H_{\pm }t+\theta_{n,\pm }\left(0\right).$$

Equation (17) is the wave functions of the system in Fock states. For further development of the wave functions to obtain a more detailed form, refer to the latter part of Appendix B. These wave functions are necessary for investigating various quantum properties of the flux qubit system coupled to the oscillator, and can be extended to more generalized states such as coherent, squeezed, and thermal states. For instance, we can manage lots of quantum characteristics, such as quadrature fluctuations, Schrödinger–Robertson uncertainty, von Neumann entropy, purity of the state, Wigner distribution function, phase properties, and transition probabilities, by means of such wave functions.

In particular, the wave functions, Equation (17), shown with phases can be practically applied to clarify pure dephasing [38,39] and its relevant concerns in superconducting qubits. Not only the high fidelity in qubit readout, long coherence time in two-level systems is also necessary. The off-diagonal density matrix elements decay during pure dephasing, while the diagonal elements are almost not affected. The understanding and addressing the underlying mechanism of such a dephasing phenomenon are theoretical challenges, vital for developing decoherence-protected quantum computing systems.

## 4. Conclusions

Quantum features of a flux qubit coupled to a harmonic oscillator have been investigated with emphasis on energy-level splitting in the oscillator. Because the Hamiltonian of the system is a complicated form, the mathematical treatment of the system in the quantum domain is not an easy task. This is the reason why many researchers relied on numerical analyses using a rotating wave approximation, instead of analytical analyses, in solving quantum problem of a qubit system coupled to an oscillator so far [40,41].

However, we have managed the system analytically in this work for the availability of detailed analyses. We have used the unitary transformation approach as a special mathematical technique for treating the matrix Hamiltonian of the system. These procedures enabled us to examine the quantum characteristics of the system in detail conforming to quantum wave mechanics. The wave functions that are requisite as the basic tools for unfolding quantum dynamics of the system were derived. Various quantum properties of the qubit systems, such as purity of the states and the von Neumann entropy, can be addressed by making use of quantum information theory that is based on these wave functions.

We have analyzed the upper ($E_{n,+}$) and lower ($E_{n,-}$) energy levels of the qubit–oscillator and the difference between them, $E_{n,d}$. The overall energy levels $E_{n,\pm }$ are composed of three terms, which are resonator energies ($E_{n,\pm }^{\mathrm{SHO}}$), qubit energies ($E_{\pm }^{q}$), and the additional energies ($E_{\pm }^{\mathrm{add}}$) that appear due to the coupling of the qubit on the resonator. Owing to such a coupling, the angular frequency $\omega_{p}$ of the resonator splits into two parts which are $\omega_{p,+}$ and $\omega_{p,-}$. As a consequence, each resonator energy level splits as $E_{n}^{\mathrm{SHO}}\to E_{n,\pm }^{\mathrm{SHO}}$. The appearance of this splitting enables us to detect qubit states through inspecting the bifurcation. Thus, the determination of qubit states utilizing such a conceptual idea for dispersive measurement is possible.

We have compared $E_{n,\pm }$ with the qubit energy $E_{\pm }^{q}$: $E_{n,\pm }$ decrease when $L_{t}$ and/or $C_{\mathrm{sh}}$ increase, meanwhile $E_{\pm }^{q}$ are irrelevant to such parameters. Roughly speaking, the energy levels with the lowest quantum number for the combined system, $E_{0,+}$ ($E_{0,-}$), is larger than the qubit energy level, $E_{+}^{q}$ ($E_{-}^{q}$), by an amount of zero-point energy of the resonator. The additional energies $E_{\pm }^{\mathrm{add}}$ are very small enough that they can be negligible in the case of the weakly coupled system. However, $E_{\pm }^{\mathrm{add}}$ strongly depend on the coupling strength λ. We have confirmed that $E_{n,d}$ are smallest when ε=0 and increase as ε grows. $E_{n,d}$ also increase as Δ grows.

The information developed here can be usefully applied in designing both a flux qubit and a readout system of qubit signals. Though we adopted the flux qubit coupled to the SQUID, our development can be tailored for the purpose of describing other similar systems and structures, such as readout mechanisms for other types of qubits and the interaction of radiation with superconducting rings [18,42,43,44].

## 5. Methods

We first introduced a Hamiltonian that describes a flux qubit system coupled to a SQUID oscillator. The Schrödinger equation associated with this Hamiltonian was solved using the unitary transformation method. The unitary transformation of the Hamiltonian was performed in two steps. The first transformation was carried out with the choice of the unitary operator as Equation (8), and then, in performing the second transformation, we used the operator given in Equation (9).

From the first transformation, the Hamiltonian matrix was diagonalized as shown in Equation (A1). However, the evaluation of the Schrödinger equation in this transformed system in a straightforward way is still difficult due to the fact that the transformed Hamiltonian involves a linear term of $\hat{q}$. This is the reason why we have secondly transformed the Hamiltonian to be a further simplified one. The final Hamiltonian derived via these two transformations is just the combination of two simple harmonic oscillators (see Equation (14)). Hence, we have easily identified the Schrödinger solutions in the transformed system as shown in Equation (A4). The full wave functions in the original system were obtained by transforming these solutions inversely (see Equation (17)). We have managed the system from a quantum-mechanical point of view throughout the paper.

## Data Availability

Not applicable.

## Appendix A. Unitary Transformation

We represent the unitary transformation, which is necessary for analyzing the energy levels of the system together with obtaining the quantum wave functions. We first transform the Hamiltonian in Equation (2) as ${\hat{H}}^{\prime }=\hat{U}\hat{H}{\hat{U}}^{†}$ where $\hat{U}$ is shown in Equation (8). A rigorous evaluation gives a diagonalized Hamiltonian of the form

(A1) $${\hat{H}}^{\prime }=\left(\begin{array}{cc} {\hat{H}}_{+}^{\prime } & 0\\ 0 & {\hat{H}}_{-}^{\prime } \end{array}\right),$$

where ${\hat{H}}_{\pm }^{\prime }=\hat{X}\pm \sqrt{{\hat{Y}}^{2}+D^{2}}.$

By employing Equation (13), the square root which appeared in the elements of Equation (A1) is approximated to be

(A2) $$\sqrt{{\hat{Y}}^{2}+D^{2}}≃\frac{\hbar \omega_{L}}{2}+\frac{2\Omega \lambda^{2}}{\hbar^{2}}\left(\frac{1}{\omega_{L}}-\frac{\varepsilon^{2}}{\omega_{L}^{3}}\right){\hat{q}}^{2}-\sqrt{\frac{2\Omega }{\hbar }}\frac{\varepsilon }{\omega_{L}}\lambda \hat{q}.$$

Here, we have regarded the terms only up to $\lambda^{2}$. If we use Equation (A2), the elements of Equation (A1) become

(A3) $${\hat{H}}_{\pm }^{\prime }≃\frac{{\hat{p}}^{2}}{2L_{t}}+\frac{1}{2}L_{t}\omega_{p,\pm }^{2}{\hat{q}}^{2}\mp \sqrt{\frac{2Ω}{\hbar }}\frac{\varepsilon }{\omega_{L}}\lambda \hat{q}\pm \frac{\hbar \omega_{L}}{2}.$$

Equation (A3) is the same as the Hamiltonian of a forced harmonic oscillator because the linear term involving $\hat{q}$ represents a force in the equation of motion [45]. The Hamiltonian can be more simplified by an additional transformation that removes such a force term. Hence, for further simplification, we use another unitary operators ${\hat{U}}_{\pm }$ shown in Equation (9). Then, by performing the second step of transformation in a way that ${\hat{H}}_{\pm }^{\prime \prime }={\hat{U}}_{\pm }{\hat{H}}_{\pm }^{\prime }{\hat{U}}_{\pm }^{†},$ we finally have Equation (14) in the text.

## Appendix B. More Detailed Derivation of the Wave Functions

From the Schrödinger equation for the final transformed Hamiltonian (Equation (14)), $i\hbar \partial \psi_{n,\pm }^{\prime \prime }\left(q,t\right)/\partial t={\hat{H}}_{\pm }^{\prime \prime }\psi_{n,\pm }^{\prime \prime }\left(q,t\right)$, the wave functions associated with ${\hat{H}}_{\pm }^{\prime \prime }$ are easily identified to be

(A4) $$\psi_{n,\pm }^{\prime \prime }\left(q,t\right)=\phi_{n,\pm }^{\prime \prime }\left(q\right)\exp\left[i\theta_{n,\pm }\left(t\right)\right],$$

where

(A5) $$\phi_{n,\pm }^{\prime \prime }\left(q\right)={\left(\frac{\Omega_{\pm }}{\hbar \pi }\right)}^{1/4}\frac{1}{\sqrt{2^{n}n!}}H_{n}\left[{\left(\frac{\Omega_{\pm }}{\hbar }\right)}^{1/2}q\right]\exp\left(-\frac{\Omega_{\pm }}{2\hbar }q^{2}\right),$$

while $\Omega_{\pm }=L_{t}\omega_{p,\pm }$ and $\theta_{n,\pm }\left(t\right)$ are phases given in Equation (19) in the text. Although the wave functions, Equation (A4), just belong to the SHO, their phases, Equation (19), are not the same as those of the SHO because of the additional term, $-\hbar^{-1}H_{\pm }t$, which remains so long as $H_{\pm }$ in Equation (14) do not vanish. From an inverse transformation of the wave functions in Equation (A4), it is possible to obtain the complete wave functions in the original system.

The wave functions in the first transformed system, $\psi_{n,\pm }^{\prime }\left(q,t\right)$, can be derived from the inverse transformation of the form $\psi_{n,\pm }^{\prime }\left(q,t\right)={\hat{U}}_{\pm }^{†}\psi_{n,\pm }^{\prime \prime }\left(q,t\right).$ A straightforward mathematical procedure for this transformation gives

(A6) $$\psi_{n,\pm }^{\prime }\left(q,t\right)=\phi_{n,\pm }^{\prime }\left(q\right)\exp\left[i\theta_{n,\pm }\left(t\right)\right],$$

where $\phi_{n,\pm }^{\prime }\left(q\right)$ are obtained from the relation $\phi_{n,\pm }^{\prime }\left(q\right)={\hat{U}}_{\pm }^{†}\phi_{n,\pm }^{\prime \prime }\left(q\right)$, while their formulas are given in Equation (18).

We now consider the next step of the inverse transformation

(A7) $$\psi_{n}\left(q,t\right)={\hat{U}}^{†}\psi_{n}^{\prime }\left(q,t\right).$$

The outcome of Equation (A7) is identical to Equation (17) in the text under the arrangement

(A8) $$\psi_{n}^{\prime }\left(q,t\right)=\left(\begin{array}{c} \psi_{n,+}^{\prime }\left(q,t\right)\\ \psi_{n,-}^{\prime }\left(q,t\right) \end{array}\right),$$

where $\psi_{n,\pm }^{\prime }\left(q,t\right)=\phi_{n,\pm }^{\prime }\left(q\right)\exp\left[i\theta_{n,\pm }\left(t\right)\right]$.

A further evaluation of Equation (A7) (or Equation (17) in the text) yields a more detailed form of the wave functions. That is, a strict evaluation of it using Equation (8) results in

(A9) $$\psi_{n}\left(q,t\right)=\left(\begin{array}{c} W\psi_{n,+}^{\prime }\left(q,t\right)+Z\psi_{n,-}^{\prime }\left(q,t\right)\\ Z\psi_{n,+}^{\prime }\left(q,t\right)-W\psi_{n,-}^{\prime }\left(q,t\right) \end{array}\right),$$

where

(A10) $$W=\frac{Y+\sqrt{Y^{2}+D^{2}}}{\sqrt{N}},\;\;\;\;\;\;\;\;\;\;\;\;Z=\frac{D}{\sqrt{N}}.$$

By comparing Equation (A9) with Equation (7), we see that

(A11) $$\psi_{n,+}\left(q,t\right)=W\psi_{n,+}^{\prime }\left(q,t\right)+Z\psi_{n,-}^{\prime }\left(q,t\right),$$

(A12) $$\psi_{n,-}\left(q,t\right)=Z\psi_{n,+}^{\prime }\left(q,t\right)-W\psi_{n,-}^{\prime }\left(q,t\right).$$

It may be possible to expand W and Z in terms of λ. We first expand *N* in terms of λ as a preliminary task before executing such expansions. Then, under the condition represented in Equation (13), *N* is given by

(A13) $$N=A_{0}+A_{1}\lambda q+A_{2}\lambda^{2}q^{2}+\cdots ,$$

where

(A14) $$A_{0}=\frac{\hbar^{2}}{2}\left(\varepsilon^{2}+\delta^{2}-\varepsilon \omega_{L}\right),$$

(A15) $$A_{1}=2\sqrt{2\Omega \hbar }\left[\frac{1}{2}\left(\omega_{L}+\frac{\varepsilon^{2}}{\omega_{L}}\right)-\varepsilon \right],$$

(A16) $$A_{2}=\frac{2\Omega }{\hbar }\left[2-\frac{\varepsilon }{\omega_{L}}\left(3-\frac{\varepsilon^{2}}{\omega_{L}^{2}}\right)\right].$$

Using this, the coefficients appearing in Equation (A9) are evaluated up to the second order of λ as

(A17) $$W=\frac{1}{\sqrt{A_{0}}}\left[B_{0}+B_{1}\lambda q+B_{2}\lambda^{2}q^{2}+O\left(\lambda^{3}\right)\right],$$

(A18) $$Z=\frac{D}{\sqrt{A_{0}}}\left[1-\frac{A_{1}}{2A_{0}}\lambda q-\left(\frac{A_{2}}{2A_{0}}-\frac{3A_{1}^{2}}{8A_{0}^{2}}\right)\lambda^{2}q^{2}+O\left(\lambda^{3}\right)\right],$$

where

(A19) $$B_{0}=\frac{\hbar }{2}\left(\omega_{L}-\varepsilon \right),$$

(A20) $$B_{1}=\left(\sqrt{\frac{2\Omega }{\hbar }}\frac{1}{\omega_{L}}-\frac{A_{1}\hbar }{4A_{0}}\right)\left(\omega_{L}-\varepsilon \right),$$

(A21) $$B_{2}=\frac{2\Omega }{\hbar^{2}}\left(\frac{1}{\omega_{L}}-\frac{\varepsilon^{2}}{\omega_{L}^{3}}\right)-\left[\frac{A_{1}}{2A_{0}\omega_{L}}\sqrt{\frac{2\Omega }{\hbar }}+\frac{\hbar }{2}\left(\frac{A_{2}}{2A_{0}}-\frac{3A_{1}^{2}}{8A_{0}^{2}}\right)\right]\left(\omega_{L}-\varepsilon \right).$$

Though we have truncated the terms higher than the second order of λ in Equations (A17) and (A18), it is also possible to increase the precision of them as much as we desire by adding more higher terms through a rigorous evaluation.
